# Supplementary material for: Changes in the Microbial Community Diversity of Oil Exploitation
Source: Genes (Basel). 2019 Jul 24;10(8):556. doi: 10.3390/genes10080556 (PMC6723437; doi:10.3390/genes10080556)
Supplement: Supplementary file 1 [file genes-10-00556-s001.pdf]

Regional changes in microbial community diversity in oil reservoir

Jingjing Liu [1,#], Jing Wu [1,#], Jiawei Lin[1,#], Jian Zhao[1], Tianyi Xu[1], Qichang Yang[1], Jing Zhao[3,*], Zhongming Zhao[4, 5, *], and Xiaofeng Song[1,*]

1 Department of Biomedical Engineering, Nanjing University of Aeronautics and Astronautics, Nanjing 210016, China

2 School of Biomedical Engineering and Informatics, Nanjing Medical University, Nanjing, Jiangsu, 211166, China

3 Dalian Chivy Biotechnology Limited Company, Dalian, 116023, Liaoning

4 Center for Precision Health, School of Biomedical Informatics, The University of Texas Health Science Center at Houston, Houston, TX 77030, USA

5 Human Genetics Center, School of Public Health, The University of Texas Health Science Center at Houston, Houston, TX 77030, USA

The first 3 authors should be regarded as joint First Authors

* Correspondence: zhaojing00815@126.com; zhongming.zhao@uth.tmc.edu; xfsong@nuaa.edu.cn

Figure S1. Taxonomic distribution of the bacterial communities in different samples at the class level within Protebacteria phylum.

Figure S2. Taxonomic distribution of the bacterial communities in different samples at the class level within Firmicutes phylum.

Figure S3. Taxonomic distribution of the bacterial communities in different samples at the class level Actinobacteria.

Figure S4. Taxonomic distribution of the bacterial communities in different samples at the class level within Acidobacteria phylum.

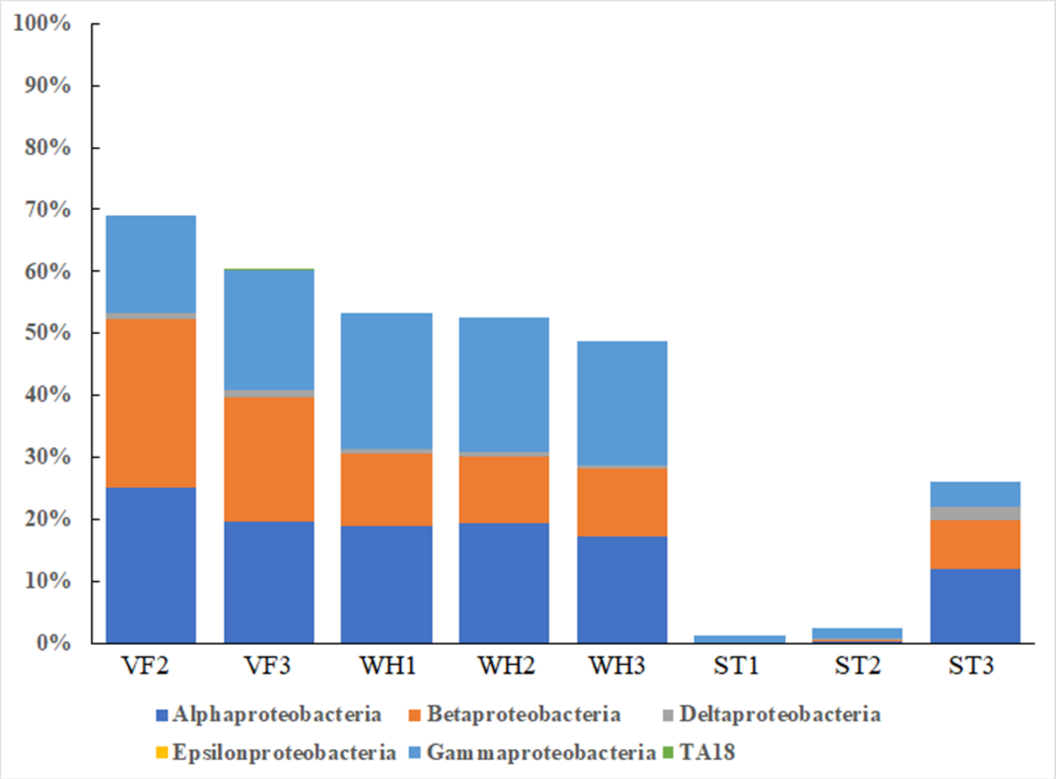

Figure S1. Taxonomic distribution of the bacterial communities in different samples at the class level within Protebacteria phylum.

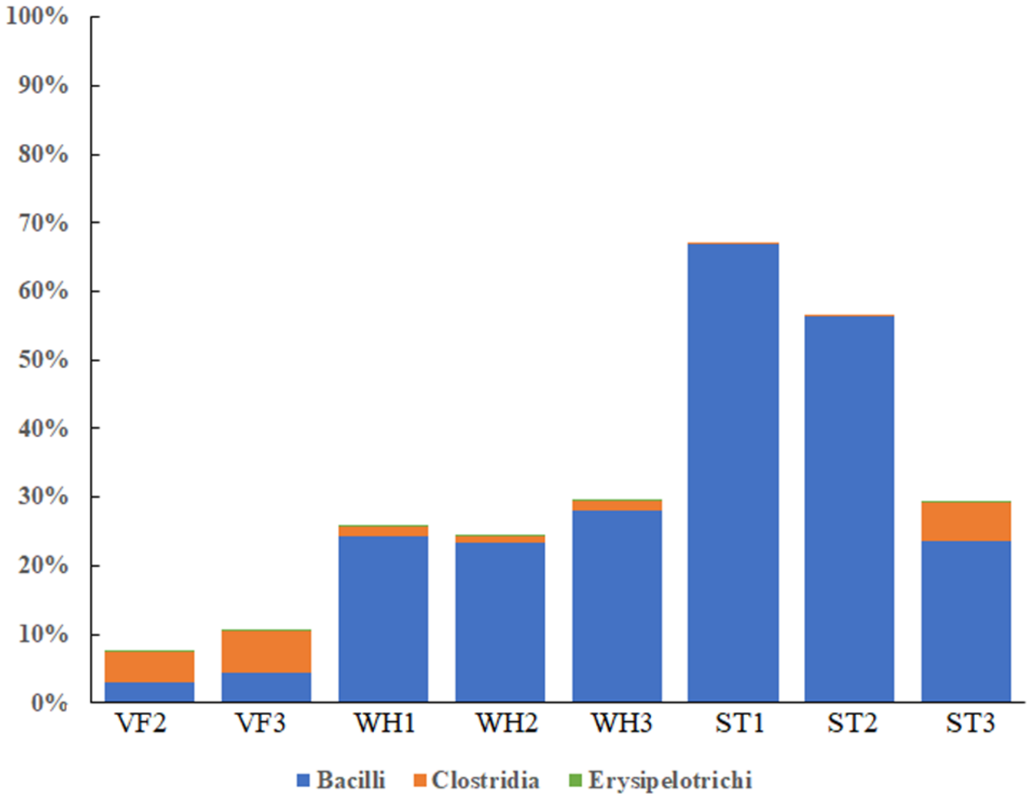

Figure S2. Taxonomic distribution of the bacterial communities in different samples at the class level within Firmicutes phylum.

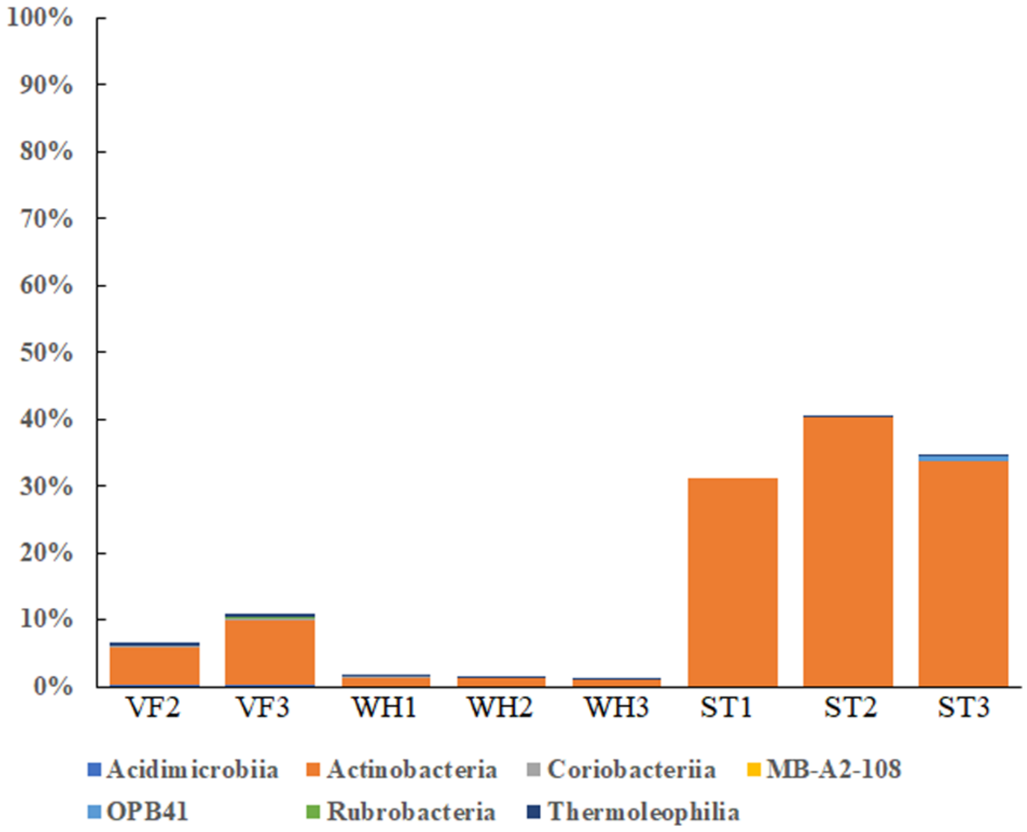

Figure S3. Taxonomic distribution of the bacterial communities in different samples at the class level Actinobacteria.

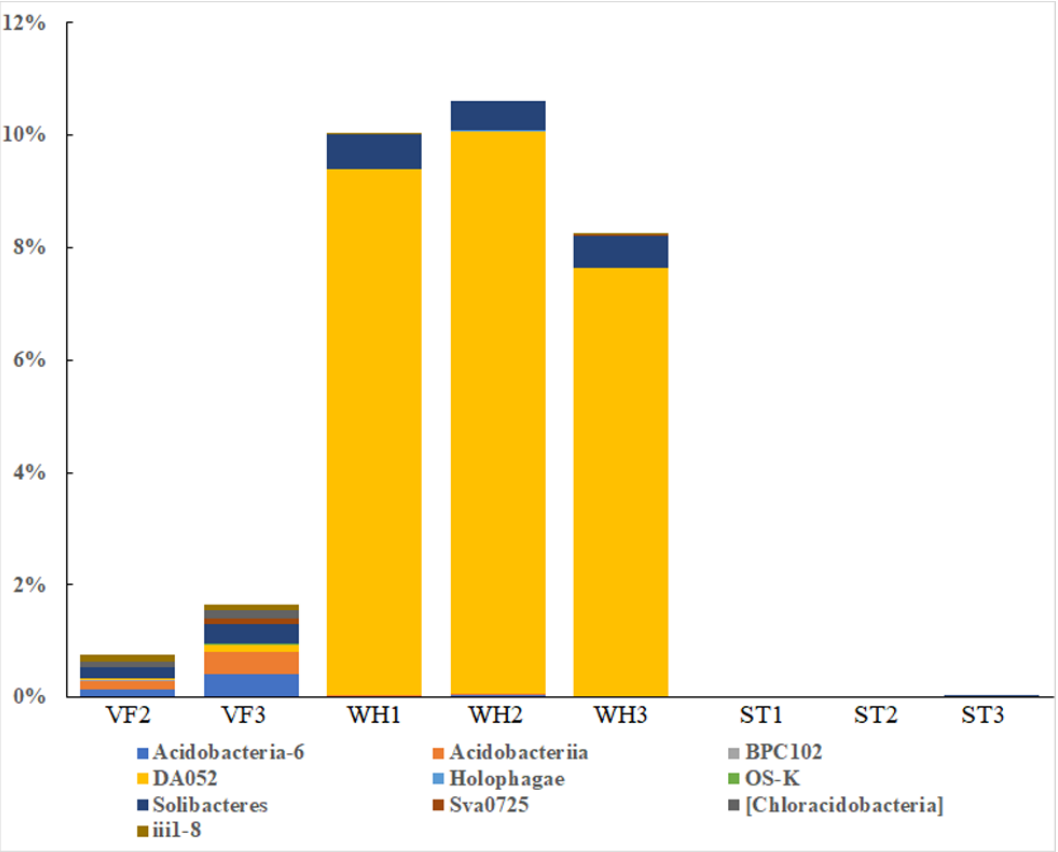

Figure S4. Taxonomic distribution of the bacterial communities in different samples at the class level within Acidobacteria phylum.
